# Comparative response of Desmopressin versus Combination Therapy (Desmopressin + Oxybutynin) in Children with Nocturnal Enuresis

**DOI:** 10.12669/pjms.36.6.1957

**Published:** 2020

**Authors:** Asiya Kazi, Kemchand N Moorani, Shabih Zehra, Ijaz Hussain Zaidi

**Affiliations:** 1 Dr. Asiya Kazi, MBBS, Pharmacology Department, Bahria University Medical & Dental College, Sailors Street, Adjacent PNS Shifa, Defence Phase 2, Karachi, Pakistan; 2 Prof. Dr. Khemchand N. Moorani, MBBS, MCPS, FCPS. Department of Paediatric Nephrology, National Institute of Child Health and Human Development (NICHD), Karachi, Pakistan; 3 Dr. Shabih Zehra, MBBS, MCPS, FCPS. Department of Radiology, PNS Shifa Hospital DHA, Karachi, Pakistan; 4 Prof. Dr. Ijaz Hussain Zaidi Faisal, MBBS, PhD. Pharmacology Department, Bahria University Medical & Dental College, Sailors Street, Adjacent PNS Shifa, Defence Phase 2, Karachi, Pakistan

**Keywords:** Nocturnal Enuresis, Desmopressin, Oxybutynin, Recurrence

## Abstract

**Objective:**

To assess the safety as well as efficacy of desmopressin monotherapy alone and in combination (desmopressin + oxybutynin) in treating nocturnal urinary incontinence among children with 7 to 13 years.

**Methods:**

This randomized controlled trial has been carried out in National Institute of Child Health from September 2018 to March 2019 with the utilization of convenient sampling technique. Data has been collected after taking ethical approval and informed consent of the Parents with complete confidentiality. The sample size was 84 and equal number of patients was divided in two groups. Group-I was given desmopressin at monotherapy at a dose of 0.2 mg and Group-II was given desmopressin and oxybutynin at the dose of 0.2 mg desmopressin and 5 mg oxybutynin patients were diagnosed on the basis of history. Routine lab investigation included Urine DR and ultrasound abdomen.

**Results:**

In this study significant differences between two groups were found with respect to socio economic status, lack of education of parents (P Less than 0.05). The frequency, urgency and incontinence of this ailment was significantly controlled by combination therapy (desmopressin + oxybutynin) as compared to desmopressin as monotherapy (P Less than 0.05) as patient was followed after one, two and three monthly basis.

**Conclusion:**

Desmopressin combination with oxybutynin is more effective as compared to monotherapy treatment. The affectivity of the combination therapy was very high with least side effects and all the children recovered from the condition at third month of treatment. Furthermore, headache was observed to be common with monotherapy and loss of appetite was observed with combination therapy.

## INTRODUCTION

Nocturnal enuresis (NE) is defined as the involuntary urination during sleep. Commonly known as bed-wetting, nocturnal enuresis occurs in night at bed during sleep WHO guidelines consider NE to be a pathological disorder if it persists after five years of age. NE causes significant inconvenience to the children and their parents. The prevalence of NE, varies according to age group and decreases as age advances, it is 15-20% at the age of 5, 5% at the age of 10, 1-2% at the age of 15 years. The primary etiology of NE is still unknown; however, the decrease in the capacity of the urinary bladder increase urine production and deep sleep are the possible explaination.^1^

The mode of inheritance of the disease is autosomal dominant with 90% penetrance, which means that the children of one parent suffering from NE have 40% chance of developing the disorder and if both parents have suffered from enuresis then this percentage is raised up to 70%. The genes related to this disorder are located on chromosomes 8, 12, 13, and 22.^2^ Furthermore, the patients of NE do not have neurological disorders, congenital or acquired; rather the change in the capacity of bladder and formation of urine during the night appear to be the primary reason of the condition.^3^

Primary nocturnal enuresis (PNE) is when a child continues to have involuntary micturition in bed without ever having developed continence. If, it occurs after a child has achieved urinary continence, it is called secondary nocturnal enuresis (SNE). Therefore, it is essential to exclude secondary causes. A through history, examination and urine analysis as well u/s KUB are mandatory to exclude urinary tract infection diabetes, neurogenic bladder and VUR.^4^

Treatment plans can be suggested in children above six years of age as the disorder is inconvenient. Usual management plans would include both medicine and also behavioral therapy. Sometimes, the management would comprise of multiple treatment modalities. If mono-therapy has proved to be ineffective then it is advisable to start combined therapies which are proven to be more effective. These therapies might contain one-to-one counseling, positive reinforcement and alarm.^5^ However, such treatment plans must be tailored case-by-case keeping in mind the needs of the child and the resources corresponding to the family’s affordance.^6^

It is also important to differentiate between primary and secondary nocturnal enuresis since in the latter case, the disorder can often be managed by treating the secondary cause.^7^ The secondary causes of NE include diabetes insipidus, diabetes mellitus, constipation, intestinal blockage, urinary tract infection, seizures, neurogenic bladder or side effects of medication.

To begin the treatment, first a comprehensive history is taken and a thorough physical examination is performed. The points to be kept in mind while taking the history are: pattern of micturition i.e. urinary frequency during day and night, incontinence during the day, and a nocturnal enuresis frequency during one week, amount of water consumed during the day, evening and before sleeping should be documented as well. After that, general physical examination is conducted. This should always include close genital examination and special neurological examination.

Among the tests recommended generally are 1) Urinalysis for urinary tract infection or glycosuria, 2) Urinary system sonography, and 3) Residual Urine measurement post-voiding. Post-void volume helps in determining the urinary bladder function. Once the diagnosis of NE has been established, method of therapy is chosen.

Buzzer alarm, behavioral modification, and medicinal treatment, are the major methods of therapy for bed-wetting. The drugs used for treatment are the ADH-analog, desmopressin, Tricyclic antidepressants (TCAs), and anticholinergic drugs, like oxybutynin.^8^ The drug of choice for PNE is considered to be desmopressin which is available in the form of pills and nasal spray. The oral route of administration is preferred as it has faster onset of action and fewer side effects. Recommended dose of desmopressin is 20µg daily (for nasal inhalation) or 0.2 mg (for oral route).^9^ Recommendations suggest desmopressin treatment to continue for 3 months. If the patient fails to improve then the disorder is called desmopressin resistant nocturnal enuresis, which occurs in 20% of cases.^10^

Unfortunately, no study predicting the use of desmopressin for nocturnal enuresis has been performed in our country. Our study is the first major study with a large sample size to validate the results of previous studies in our population. This will help clinicians in making informed choice about the management plan and increase awareness about the safety and efficacy of desmopressin and oxybutynin in our country. Therefore, the objective of our study was to evaluate the safety and efficacy of desmopressin monotherapy and its comparison with combination therapy with oxybutynin.

## METHODS

This study was randomized controlled trial and convenient sampling technique was used to select the sample. The study was conducted at Bahria University Medical & Dental College (PNS Shifa Hospital) and the National Institute of Child Health, Karachi from September 2018 to March 2019 after obtaining ethical approval from the Ethical Review Committee (IERB No: 16/2017). Data was collected from a sample size of 84 children after taking informed consent in writing from the parents and/or any other guardian of each child, ensuring thorough confidentiality. Eighty Four patients were divided into two groups of 42 children each labeled as Group-I and Group-II. Group-I comprised of children on desmopressin therapy alone and Group-II comprised of children receiving a combination therapy of desmopressin and oxybutynin. The required sample size in each Group-I.e. n1=42, n2=42, total= 84 was calculated using the software G POWER 3.1.9.2 with two portions; desmopressin monotherapy = 72.4% and desmopressin + oxybutynin = 83.3% taken from the study. The efficacy of desmopressin and oxybutynin combination therapy in children with nocturnal enuresis was compared via a randomized clinical trial using confidence interval for mean at 95% while keeping a 5% margin of error. Patients of either gender between the age group of 7 to 13 years who were diagnosed cases of nocturnal enuresis on the basis of clinical findings and history were included in the study. Patients with congenital anomalies, seizures and abnormalities of the central nervous system, diabetes insipidus, urinary tract infections or with a history of any other co morbids were excluded from the study. Subjects who were non-compliant or unable to follow up through the complete study period and patients whose parents/guardians did not consent for inclusion were also excluded.

Group-I comprised of patients on desmopressin monotherapy where a daily dosage of 0.2mg of desmopressin was given once daily for 90 days. Group-II had patients on combination therapy of desmopressin (0.2mg OD) orally + oxybutynin (5mg OD for 90 days). Double blinding of the study was done with neither the patients nor the doctors being aware of the patients on drug monotherapy or combination therapy. Patients were examined on the first visit and diagnosed on the basis of their history. Each patient was followed at the end of 1^st^, 2^nd^ and 3^rd^ month. After recording the demographic variables, the primary outcome was observed in terms of partial recovery, complete recovery and no recovery. Then, the patients were categorized into mild (once per week), moderate (two times per week) and severe (one time daily) nocturnal enuresis The safety of the drug and its combination therapy was assessed as the occurrence or absence of various side effects observed by the patient during the study period which included headache, nausea, vomiting, confusion, weakness, weight gain and/or loss of appetite.

### Sample Size & Statistical Analysis

Sample size was calculated by two proportion method. n=P1-P2/Square root [{P1(1-P1)/n1} + {P2(1-P2)/n2}]. SPPS software version 20.0 was used to statistically analyze the data. Qualitative data was expressed as frequency and percentage and quantitative data was stated as mean and standard deviation, with independent t-test being used to calculate the mean difference. Chi-square test was applied to assess the association with p-value of <0.05 as the significant level.

## RESULTS

Sample size was kept at a total of 84 children with 42 children in each group. In a total of 84 patients divided into two groups, the mean age of the patients was observed to be 9.30±1.89 years in Group-I and 8.57±2.24 years in Group-II with insignificant difference (p=0.250). Mean weight of the patients with a significant difference (p=0.010) was observed with 22.80±7.33 kg in Group-I and 21.28±4.53 kg in Group-II. Both the groups had similar educational status - 97.6% with primary level of education and 2.4% with middle. Insignificant association was observed in the socioeconomic class between the two groups (p=0.359). 11.9% of the patients in Group-I presented with a family history of hypertension whereas 4.8% of the patients in Group-II with insignificant association (p=0.236). 2.4% of the patients in Group-I and 4.8% of the patients in Group-II with insignificant association (p=0.557) had a family history of diabetes. Both the groups had monosymptomatic type of nocturnal enuresis. 7.1% of the patients in Group-I and 2.4% of the patients in Group-II had a family history of nocturnal enuresis on the maternal side with insignificant association (p=0.306). Only 2.4% of the cases in both the groups had a family history of nocturnal enuresis on the paternal side. 61.9% of the patients in Group-I and 69.0% in Group-II patients with insignificant association (p=0.491) had siblings with a history of nocturnal enuresis. None of the cases from either of the groups presented with complain of day time wetting and/or irregularities in bowel habits.

7.1% of the patients showed severity of mild nature, 35.7% showed moderate and 57.1% exhibited extremely severity in Group-I. However, in Group-II, 2.4% of the patients were mild, 31.0% were moderate and 66.7% were severe with insignificant association (p=0.484).

**Table-I:** Educational status of the children in different groups.

Variables			Group-I	Group-II	P-Value

			n(%)	n(%)	
Educational Status	Primary		41(97.6%)	41(97.6%)	--
	Middle		1(2.4%)	1(2.4%)
Socioeconomic Status	Upper		1(2.4%)	0 (0.0%)	0.359
	Lower Middle		1 (2.4%)	0 (0.0%)
	Lower		40 (95.2%)	42 (100.0%)
Family History of comorbids	Hypertension	Yes	5 (11.9%)	2 (4.8%)	0.236
		No	37(88.1%)	40(95.2%)	
	Diabetes	Yes	1 (2.4%)	2(4.8%)	0.557
		No	41(97.6%)	40(95.2)	
Family History of nocturnal enuresis	Mother	Yes	3 (7.1%)	1 (2.4%)	0.306
		No	39(92.9%)	41 (97.6%)	
	Father	Yes	1 (2.4%)	1 (2.4%)	1.000
		No	41(97.6%)	41(97.6%)	
	Siblings	Yes	26(61.9%)	29(69.0%)	0.491
		No	16(38.1%)	13(31.0%)	

Group-I: Desmopressin, Group-II: Desmopressin + oxybutynin,

P value < 0.05 = Significant, Test: Chi-square.

At the 4^th^ week, in Group-I, 13(31.0%) showed complete recovery, 27(64.32%) showed partial recovery and two (4.8%) showed no recovery. Similarly, 13(31.0%) showed complete, 29(69.0%) showed partial and 0 (0.0%) showed no recovery in Group-II with insignificant association between the groups (p=0.355). At the 8^th^ week 28(66.7%) patients showed complete and 14(33.3%) patients showed partial recovery in Group-I. Similarly, 33(78.6%) patients showed complete and 9 (21.4%) showed partial recovery in Group-II with insignificant association (p=0.221)

**Table-II:** Severity of bed wetting at baseline in children of different groups.

Variables		Group-I	Group-II	P-Value

		n(%)	n(%)	
Severity of bed wetting (At Baseline)	Mild	3 (7.1%)	1 (2.4%)	0.484
	Moderate	15(35.7%)	13(31.0%)	
	Severe	24(57.1%)	28(66.7%)	
Recovery Status at (4^th^ week)	Complete Recovery	13(31.0%)	13(31.0%)	0.355
	Partial Recovery	27(64.32%)	29(69.0%)	
	No Recovery	2 (4.8%)	0 (0.0%)	
Recovery Status at ( 8^th^ week)	Complete Recovery	28(66.7%)	33(78.6%)	0.221
	Partial Recovery	14(33.3%)	9 (21.4%)	
	No Recovery	0 (0.0%)	0 (0.0%)	
Recovery Status at ( 12^th^ week)	Complete Recovery	36(85.7%)	42(100.0%)	0.040
	Partial Recovery	5 (11.9%)	0 (0.0%)	
	No Recovery	1 (2.4%)	0 (0.0%)	

Group-I: Desmopressin, Group-II: Desmopressin + oxybutynin,

P value Insignificant, Test: Chi-square.

**Table-III:** Side effects of treatment in children of different groups.

Variables			Group-I	Group-II	p-value

			n(%)	n(%)	
Side Effect	Allergic Reactions	Yes	7 (16.7%)	0 (0.0%)	0.006
		No	35 (83.3%)	42(100.0%)	
	Nausea	Yes	7 (16.7)	1 (2.4%)	0.026
		No	35(83.3%)	41(97.6%)	
	Loss of Appetite	Yes	2 (4.8%)	16 (38.1%)	<0.001
		No	40 (95.2%)	26 (61.9%)	
	Headache	Yes	12 (28.6%)	2 (4.8%)	0.003
		No	30 (71.4%)	40 (95.2%)	

Group-I: Desmopressin, Group-II: Desmopressin + oxybutynin,

P value Significant- Allergic reaction, Nausea, Loss of appetite, Headache, Test: Chi-square.

At the 12^th^ week 36(85.7%) patients showed complete recovery, five (11.9%) patients showed partial and one (2.4%) showed no recovery in Group-I. Whereas complete recovery was observed in all the 42 cases (100.0%) in Group-II with significant association (p=0.040)

As far as the side effects were concerned, 7 (16.7%) patients in Group-I and 1(2.4%) in Group-II developed nausea with significant association (p=0.026). Loss of appetite was observed in 4.8% of the patients in Group-I and 38.1% in Group-II with significant association (p<0.001). 12 (28.6%) patients in Group-I and 2(4.8%) patients in Group-II complained of headache, with significant association (p=0.003). 16.7% patients in Group-I exhibited allergic reactions whereas none exhibited the same in Group-II with significant association (p=0.006). Vomiting, weight gain, confusion and/or weakness were not exhibited by any of the patients in both the groups.

**Fig.1:**
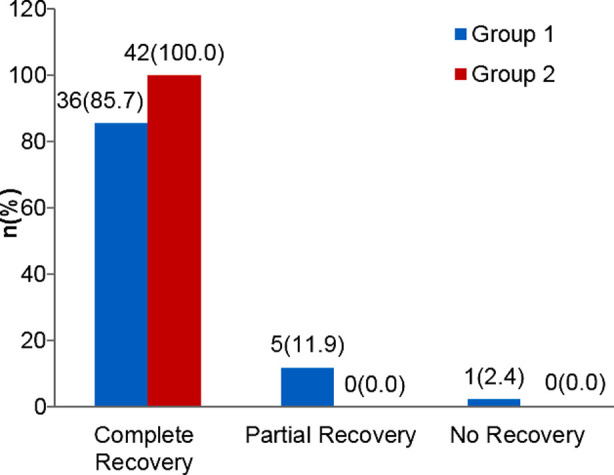
Severity of bed wetting at 12^th^week of treatment in children of different groups.

## DISCUSSION

The recommended management plans for nocturnal enuresis (NE) include behavioral therapy, alarm therapy, and drug therapy. The drugs recommended for management include desmopressin, anticholinergic drugs and tricyclic antidepressants. However, drug monotherapy has not proven to be particularly effective. Therefore, combination therapy with desmopressin is usually the treatment of choice.^11^ About 20% to 60% of patients have desmopressin resistant nocturnal enuresis. These patients have reduced bladder volume but higher urine osmolarity as compared to the patients who respond well to the desmopressin therapy. Dairy intake might increase the urine osmolarity therefore dairy intake should be restricted.^3^ Desmopressin resistant NE is also associated with irregular bowel habits, sleep disorders, and mental disorders. The resistance in patients with nocturnal enuresis might frequently correlate with the occurrence of irregular bowel habits, mental and sleep disorders. Therefore, appropriate management is necessary.^12^

A study to compare the efficacy of desmopressin monotherapy and desmopressin and oxybutynin combination therapy was performed on 59 children aged 3 to 15. After a month of treatment, the success rate was 44.8% in monotherapy and 83.3% in combination therapy. These increased to 72% and 86.7% for monotherapy and combination therapy, respectively, after three months of treatment.^13^ Other studies comparing the efficacy of these therapies have also showed higher success rate for combination therapy.^14^,^15^ Our study also reported the similar results.

In another study, the recovery rate of monotherapy with desmopressin for three months in children aged 5 to 14 years was 82.5%.^16^ This is slightly lower than the 87% recovery rate observed in our study after 3 month of monotherapy.

A study of association of efficacy of desmopressin for NE with age and gender concluded that a significant association cannot be proved. An interesting finding was the higher recovery rate in children above seven years of age. Further research is suggested to prove these assertions.^16^ A study by Radvanska et al, concluded that monotherapy with desmopressin is less effective when compared to the combination therapy with oxybutynin. Combination therapy is helpful in cases which are desmopressin resistant.^17^ In another study with control group, consisting of desmopressin with placebo, and experimental group, given desmopressin with tolterodine, the combination therapy i.e. given to the experimental group, was found to be more effective.^18^ Our study also found higher efficacy of combination therapy.

Rombis et al conducted to compare the efficacy of combination therapy of desmopressin with oxybutynin with that of desmopressin with tolterodine. It concluded that the combination of desmopressin with tolterodine was much more effective.^19^ However, our study showed the combination therapy of desmopressin with oxybutynin to be more effective.

In another study performed on 142 children with NE, the children were randomly assigned to three groups: Group-I treated with behavioral therapy, Group-II treated with desmopressin in combination with oxybutynin and Group-III treated with desmopressin in combination with tolterodine.^20^ The results of this study are in line with our study in which the combination therapy of desmopressin with oxybutynin showed higher recovery rate. Similar studies have shown behavioral therapy alone to be less effective than pharmacological therapy.^21^ The effects of desmopressin on NE can be observed immediately but the effect is more pronounced after three weeks of treatment.^22^ Our study had similar results. The symptoms of the disease are relieved much more effectively by combination therapy than by monotherapy.^23^ However, the International Children’s Continence Society (ICCS) consider NE to be a relatively benign condition and recommends desmopressin monotherapy.^24^,^25^

Martin et al.^26^, compared oxybutynin monotherapy with combination therapy of oxybutynin with desmopressin. They concluded that the combination therapy is much more effective than the monotherapy. Furthermore, the alleviation of symptoms is much higher in combination therapy as compared to monotherapy.^14^,^26^ This conclusion is also in concordance with the findings in our study.

The clinical trial conducted by Lee et al, presented a more complicated picture. It stated that while combination therapy has comparable cure rate as compared to the monotherapy, but the combination therapy requires much less time for cure. A randomized control trial was done to compare the efficacy of oxybutynin and desmopressin. It also found that the desmopressin monotherapy is more effective.^14^

As regards side-effects, the common side effects of drug-therapy are dry mouth and constipation. The frequency of these side effects is slightly higher in combination therapy, but the difference is insignificant. In our study, allergic reactions, nausea, and headache were the common side effect of monotherapy. However, loss of appetite was seen more in combination therapy. A study to assess the safety and efficacy of tolterodine monotherapy for NE found that the side effect of diarrhea was observed in 13% of the patients.^27^ Our study did not find any gastrointestinal side effects either in monotherapy or combination therapy. In our study, the most common side effects were headache for monotherapy, and loss of appetite for combination therapy. However, our study might not be free from reporting and observer bias.

### Limitations of the study

It is an experimental study. The findings of this study suggestive of associations of effectiveness of monotherapy and combination therapy might not be immune from observer and reporting bias.

## CONCLUSION

Complete response /recovery was achieved with combination therapy in all children (100%) where as it was 85.7% in children who received desmopressin as monotherapy for 12 weeks. Our study showed that the combination therapy of desmopressin with oxybutynin is more effective as compared to desmopressin alone. However, loss of appetite was common with combination therapy and headache was commonly observed with monotherapy. There were insignificant side effects observed in either group. However, appetite was observed in combination group.

### Recommendations:

Studies with longer follow ups and multiple observers who could take the multiple readings would be useful to reduce the bias in future studies and help in generalization of result.

### Authors’ Contribution:

**AK:** Principal investigator & Researcher, did data collection and manuscript writing, is responsible for integrity of research.

**KNM:** Clinical co-supervisor of the study, provided clinical expertise.

**SZ:** Performed ultrasound scan to rule out any abnormality in urinary bladder.

**IHZ:** Supervisor of the study. Conceived the idea and proof read the manuscript.
